# Wood Distillate Promotes the Tolerance of Lettuce in Extreme Salt Stress Conditions

**DOI:** 10.3390/plants13101335

**Published:** 2024-05-12

**Authors:** Riccardo Fedeli, Silvia Celletti, Stefano Loppi

**Affiliations:** 1 BioAgry Lab, Department of Life Sciences (DSV), University of Siena, 53100 Siena, Italy; riccardo.fedeli@student.unisi.it (R.F.); stefano.loppi@unisi.it (S.L.); 2 BAT Center—Interuniversity Center for Studies on Bioinspired Agro-Environmental Technology, University of Naples “Federico II”, 80138 Napoli, Italy

**Keywords:** antioxidant power, malondialdehyde, oxidative stress, pyroligneous acid, sodium chloride

## Abstract

Soil salinization is an adverse phenomenon in agriculture that severely affects crop growth and yield. The use of natural products, such as wood distillate (WD, derived from the pyrolysis of woody biomass), could be a sustainable approach to enhance the tolerance of plants cultivated in the saline soils. Hence, this study aimed to evaluate the potential of WD, a foliar sprayed at 0.2% (*v*/*v*), in lettuce plants subjected to grow under both moderate and high soil sodium chloride (NaCl) concentrations (ranging from 0 to 300 mM). The changes in the physiological and biochemical responses of these plants to the varying salt stress conditions allowed the identification of a maximum tolerance threshold (100 mM NaCl), specific to lettuce. Beyond this threshold, levels related to plant defense antioxidant power (antiradical activity) were lowered, while those indicative of oxidative stress (malondialdehyde content and electrolyte leakage) were raised, causing significant losses in leaf fresh biomass. On the other hand, WD significantly improved plant growth, enabling plants to survive high salt conditions >200 mM NaCl. Collectively, these observations highlight that treatments with WD could be of paramount importance in coping with current environmental challenges to have better yields under soil conditions of high salt concentrations.

## 1. Introduction

A soil becomes saline when it contains a high amount of dissolved salts in the soil solution, such as sodium (Na^+^), chlorine (Cl^−^), and potassium (K^+^) ions, showing a value of electrical conductivity (EC) ≥ 2 dS^−1^ m^−1^ [1]. This excessive concentration of salts can have a negative impact on plant growth and development, both physiologically and morphologically [2]. Basically, a high salt concentration in soil is harmful to plants because it interferes with the normal activity of leaf transpiration and root water uptake, causing osmotic and ionic stress [3]. As a result, plants can show symptoms such as thick leaves with the accumulation of Na^+^ and Cl^−^ within the cells, wilting, discoloration, premature aging, and leaf drop, as well as slowed or even inhibited growth [4]. High salt concentrations in the external medium can induce the closure of leaf stomata; this is a defense mechanism activated by plants, which, in turn, can cause oxidative stress due to the transfer of excess energy to oxygen (O_2_), resulting in the formation of superoxide (O2^•−^), hydrogen peroxide (H_2_O_2_), hydroxyl radical (OH^•^), and singlet oxygen (^1^O_2_) [5,6]. These molecules are highly reactive O_2_-containing molecules (known as reactive oxygen species—ROS—) and, when in excess, they are harmful to the life of cells [7]. As a result, plants activate a series of antioxidant enzymes and biosynthesize different non-enzymatic antioxidant compounds to eliminate excess ROS or convert ROS into less toxic compounds [8,9]. Furthermore, high salt levels cause not only plant oxidative stress, but also lead to a great accumulation of electrolytes (e.g., Na^+^, Cl^−^, K^+^) within plant cells; consequently, the osmotic pressure increases, leading to the rupture of cell plasma membranes and, finally, to cell death [10]. 

This can lead to severe economic losses in crop quality and yield [11]. However, the extent of damage depends not only on the concentration of salts in the soil, but also on the ability of plants to tolerate and adapt, which varies not only from species to species, but also according to the plant growth stage [12,13].

Globally, more than 800 million ha of agricultural soils are affected by salinization problems [14]. This is of concern especially in regions characterized by arid and semi-arid climates, where water evaporation is accelerated, causing surface salts to concentrate and form a saline crust on the soil surface [15,16]. Consequently, soils lose their physical, chemical, and biological fertility, becoming infertile for agriculture and are often abandoned [17]. In Europe, soil salinization is a remarkable phenomenon affecting a large area, between 1 and 3 million ha, mainly in countries around the Mediterranean basin [18]. This problem is exacerbated by climate change, which leads to higher temperatures and prolonged and intensified drought periods, along with the adoption of inappropriate irrigation farming practices [19]. This is predicted to lead to more than 50% of agricultural soils becoming uncultivable by the end of 2050 due to excess salts [20,21]. In addition, the United Nations estimated that the world population will reach 9.7 billion people by 2050, significantly increasing the demand for food, despite the decrease in usable agricultural areas [22]. This situation makes it crucial to improve crop yield and quality to limit the problem of food security and malnutrition [23,24].

To achieve the goal of improving crop tolerance to salinized conditions, mainly two approaches have been adopted: agronomic techniques and genetic selection. Agronomic techniques involve the use of specific products, such as biofertilizers, phytohormones, potassium salts, and silicon, to modify the metabolic behavior of plants so that they can better adapt to the salinized environment [25]. Alternatively, attempts have been made to genetically select crops that show a greater salt tolerance [26]. However, so far, genetic manipulation has been limited by the complexity of gene regulation, which varies significantly during the different stages of plant development. As a result, there have been just a few cases in which salt-tolerant plant varieties have been developed using these methods [27]. 

Current environmental policies globally encourage sustainable agronomic measures to address climate change, including salt stress management for plants [28]. An eco-friendly alternative to synthetic chemicals, such as pesticides and fertilizers, is wood distillate (WD), also known as pyroligneous acid or wood vinegar [29]. This dark amber-brown natural product is obtained by distillation during the pyrolysis of woody biomass [30]. Experimentation has shown that WD is both safe for the environment [31,32], human health [33], and beneficial for plant biodiversity [34]. In Italy, it has even been approved for use in organic farming [35].

In agriculture, promising results have been obtained in the use of WD as a plant corroborant and a biostimulant [36,37]. Dissolved WD contains more than 200 organic compounds including acids, alcohols, aldehydes, ketones, phenols, and minerals (mainly calcium and iron) [38]. The composition of liquid products that are obtained from thermochemical processes, such as WD in our case, obtained from pyrolysis, can be influenced by the operating parameters (mainly, temperature, residence time, and pressure) and the type of feedstock used in the process [39,40]. Specifically, while pressure affects the kinetics and pathways of pyrolysis reactions, temperature, as well as the duration of the process, have a significant impact on the characteristics of the final product; as an example, rapid heating promotes the rapid release of volatile compounds and, as a result, the composition of the product will be different than that of a product obtained at a slower heating rate. It can be applied both to the soil by fertigation and to the leaves by spraying. Its applications include improving soil quality [41], controlling weeds [42], regulating plant growth [36], and reducing the negative effects of the presence of bioplastics in the soil [38]. However, the efficacy of WD depends on the concentration used; if WD is applied between 0.2% and 0.5% (*v*/*v*), it stimulates plant growth and yield [36,38,43,44], while if it is applied at dosages higher than 0.5% (*v*/*v*), it can have herbicidal effects [42,45,46]. To the best of our knowledge, lettuce irrigated with saline water has not been the subject of WD studies.

In this context, this study aims to investigate whether WD can improve the ability of lettuce plants to tolerate moderate and high soil sodium chloride concentrations. This study provides an understanding of how lettuce plants, treated with WD as a foliar spray, tolerate saline irrigation.

## 2. Results

### 2.1. Response of the WD-Untreated Plants to Varying Salt Concentrations

**Figure 1:**
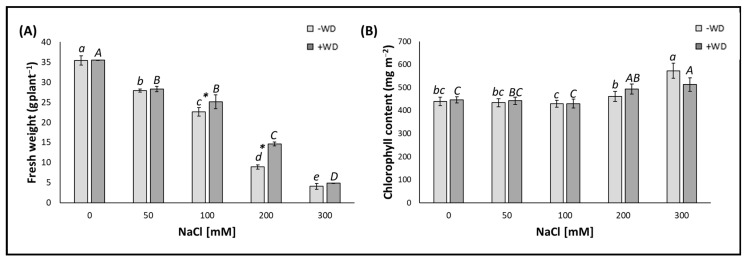
Fresh weight (**A**) and chlorophyll content (**B**) presented as the median ± error. Potting soil treatments with different NaCl concentrations [from 0 (control) to 300 mM] are displayed on the horizontal axis. “−WD” means leaves sprayed with water, while “+WD” means leaves sprayed with 0.2% (*v*/*v*) wood distillate (WD). Lowercase letters indicate significant differences (*p* < 0.05) among the different NaCl concentrations within “−WD”, whereas uppercase letters indicate significant differences (*p* < 0.05) among the different NaCl concentrations within “+WD”. *: indicates significant differences within the same salt treatments evaluated by the Kolmogorov–Smirnov test (*p* < 0.05).

The addition of salt to the soil, irrespective of the NaCl concentration, resulted in a significant reduction in leaf fresh weight of plants, which were not sprayed with 0.2% (*v*/*v*) WD (indicated as “−WD”). Specifically, the reductions increased linearly with the increasing NaCl concentration as follows: −22.1% for the 50 mM, −36.9% for the 100 mM, −75.1% for the 200 mM, and −88.6% for the 300 mM NaCl, compared to those plants which were neither treated with NaCl nor with WD (control) (Figure 1A). On the other hand, leaf chlorophyll content in the control plants did not change except at the highest NaCl concentration (300 mM), where it increased significantly (*p* < 0.05), reaching values approximately +23% higher than the control (Figure 1B).

**Figure 2:**
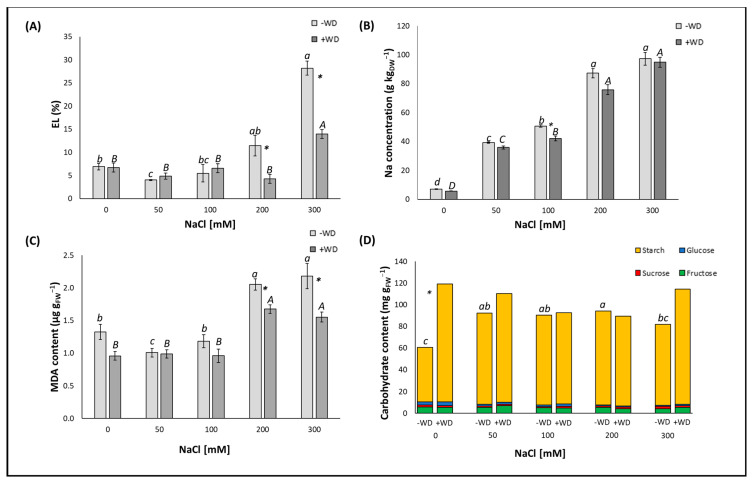
Electrolyte leakage (EL) (**A**), Na concentration (**B**), malondialdehyde (MDA) content (**C**), and carbohydrate content (i.e., glucose, fructose, sucrose, and starch) (**D**) presented as the median ± error. Potting soil treatments with different NaCl concentrations [from 0 (control) to 300 mM] are displayed on the horizontal axis. “−WD” means leaves sprayed with water, while “+WD” means leaves sprayed with 0.2% (*v*/*v*) wood distillate (WD). Lowercase letters indicate significant differences (*p* < 0.05) among the different NaCl concentrations within “−WD”, whereas uppercase letters indicate significant differences (*p* < 0.05) among the different NaCl concentrations within “+WD”. *: indicates significant differences within the same salt treatments evaluated by the Kolmogorov–Smirnov test (*p* < 0.05).

The 50 mM NaCl treatment significantly (*p* < 0.05) lowered the EL by −30%, which is an indicator of osmotic stress. Concentrations higher than 50 mM but less than 300 mM did not change this parameter, but above 300 mM NaCl, a great increase (+308.7%) in EL was observed compared to the control plants (Figure 2A). Amongst the electrolytes, however, Na accumulated abundantly in the leaves of −WD plants, increasing linearly with increasing NaCl concentrations in the following order: +453.4% (at 50 mM) < +613.5% (at 100 mM) < +1128.5% (at 200 mM) < +1269.0% (at 300 mM), with respect to the control. As an example, at 300 mM, the Na content was about 100 g kg_DW_^−1^ (Figure 2B). Similarly to EL, the content of MDA, a cytotoxic target indicative of the oxidative damage of cell membrane lipids, decreased significantly (*p* < 0.05) (−24.2%) at 50 mM. NaCl remained stable at 100 mM and then increased strongly above this concentration. Particularly, the increases were +55.3% and +65.2% for 200 and 300 mM NaCl, respectively, in comparison to the control (Figure 2C). For carbohydrates, no variation was observed between the contents of the three sugars (such as fructose, glucose, and sucrose) and the respective controls in the various treatments; instead, the starch content varied with an almost opposite trend to those of EL and MDA. In fact, the accumulation of starch increased at the lowest NaCl concentrations (at 50 mM by +71.6%; at 100 mM by +69.3%; and at 200 mM by +77.4%), whereas at the highest NaCl concentration, no change was evident when compared to the control (Figure 2D).

**Figure 3:**
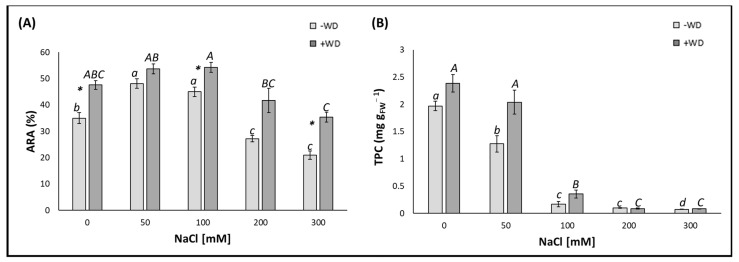
Antiradical activity (ARA) (**A**) and total phenol content (TPC) (**B**) presented as the median ± error. Potting soil treatments with different NaCl concentrations [from 0 (control) to 300 mM] are displayed on the horizontal axis. “−WD” means leaves sprayed with water, while “+WD” means leaves sprayed with 0.2% (*v*/*v*) wood distillate (WD). Lowercase letters indicate significant differences (*p* < 0.05) among the different NaCl concentrations within “−WD”, whereas uppercase letters indicate significant differences (*p* < 0.05) among the different NaCl concentrations within “+WD”. *: indicates significant differences within the same salt treatments evaluated by the Kolmogorov–Smirnov test (*p* < 0.05).

The ARA, expressing the total antioxidant power of −WD-leaves for NaCl concentrations of 50 and 100 mM was higher than the control by +37.4% and +28.6%, respectively, while for NaCl concentrations of 200 and 300 mM, it was less than the control by −22.3% and −40.3%, respectively (Figure 3A). Differently, the antioxidant compounds, in terms of TPC, decreased progressively as the NaCl concentration increased, ranging from values of 2 mg g_FW_^−1^ (control) to approximately 0.1 mg g_FW_^−1^ for the highest NaCl concentration (Figure 3B).

### 2.2. Response of the WD-Treated Plants to Varying Salt Concentrations

The effect of NaCl on the leaves of lettuce plants sprayed with 0.2% (*v*/*v*) WD (indicated as “+WD”) was evident in terms of reducing the fresh weight of the aerial part of these plants. These reductions were around lighter losses of approximately −25% on average for the 50 mM and 100 mM NaCl concentrations and heavier losses of −58.9% and −86.2% for the respective 200 mM and 300 mM NaCl concentrations, compared to those plants which were not treated with NaCl but with WD (control) (Figure 1A). On the other hand, in the WD-treated plants, the chlorophyll content only varied in the leaves of those plants treated with NaCl at the two highest concentrations (200 and 300 mM), with increases of +11.0% and +14.6%, respectively, when compared to the control (Figure 1B).

The osmotic stress index, i.e., EL, remained stable in the WD-plants treated with NaCl concentrations between 50 and 200 mM, while above 200 mM NaCl a significant increase (*p* < 0.05) was observed, which was about twofold higher than that of the control (Figure 2A). Sodium was abundantly accumulated in the WD-treated leaves, reaching approximately 95 g at the highest NaCl concentration. As expected, the Na content increased linearly with increasing NaCl concentrations as follows: +527.0% (at 50 mM) < +633.5% (at 100 mM) < +1223.1% (at 200 mM) < +1555.8% (at 300 mM), compared to the control (Figure 2B). In the WD plants, the index of cellular oxidative damage, i.e., MDA content, exhibited a similar trend as EL. Specifically, the MDA content showed a significant increase (*p* < 0.05) at 200 mM and 300 mM (+75.8% and +63.2%, respectively), while EL showed a significant increase at 300 mM (+109.0%) (Figure 2C). The content of the analyzed carbohydrates (both simple and complex) was not affected by the combined treatment of WD and NaCl, regardless of the NaCl concentration in comparison to the respective controls (Figure 2D).

Also, in the case of the ARA of the lettuce leaves, no statistical difference was detected between the WD, treated at any NaCl concentration, and the control (Figure 3A). Looking at the TPC, all of the NaCl concentrations, except for the 50 mM, which showed no significant difference to the control, resulted in a severe decline in these antioxidant compounds: −85.3% for the 100 mM and −96.5 for both 200 and 300 mM NaCl (Figure 3B).

### 2.3. Differences in the Response between WD-Untreated and WD-Treated Plants at Various Salt Concentrations

The leaf fresh weight varied between plants without WD and with WD (indicated as “−WD” and “+WD”, respectively) for concentrations 100 and 200 mM NaCl. In the presence of WD, an increase, albeit significant (*p* < 0.05), of only +11.1% was found for the 100 mM NaCl concentration, whereas it was more pronounced (+64.0%) for the 200 mM NaCl concentration (Figure 1A). In contrast, chlorophyll content was unaffected by the presence or absence of WD spraying, under any of the specific salt stress conditions tested (Figure 1B).

A significant slowdown of EL was markedly evident with WD solely at the two highest NaCl concentrations. Precisely, at 200 mM NaCl, the reduction was −66.9% and at 300 mM NaCl, it was −50.4% (+WD vs. −WD) (Figure 2A). For the specific electrolyte, namely Na, its accumulation at the leaf level was reduced by about −17.1% with the addition of WD, exclusively at the 100 mM NaCl concentration (Figure 2B). Similar to EL, a reduction in the MDA content was observed, though to a lesser extent, upon the addition of WD to the two highest NaCl concentrations. Specifically, the reduction was −18.5% at 200 mM NaCl, and −28.9% at 300 mM NaCl, when comparing +WD to −WD (Figure 2C). While foliar spraying with WD induced no change in the content of simple carbohydrates (i.e., fructose, glucose, and sucrose) in this plant tissue, WD did allow these plants to accumulate a higher starch content in their leaves, but only when either salt stress was not imposed (+122.8) (Figure 2D).

The ARA was also enhanced by WD treatment if no NaCl was present in the medium. However, this parameter also increased if the NaCl concentration was 100 and 300 mM, showing more modest increases at 0 and 100 mM (around 30% on average for both), and more accentuated increases (of almost 70%) at 300 mM (Figure 3A). In contrast, TPC showed no significant variation between the leaves of −WD and +WD lettuce plants at any NaCl concentration considered (Figure 3B).

## 3. Discussion

Several studies have shown the important role of natural products in improving nutritional quality, growth and the yield of crop plants under several abiotic stresses, including salt stress [47,48,49,50,51,52,53,54]. Among these products, WD has proved to be very effective [36,55], although its efficacy in mitigating the damage caused by salt stress is not yet well explored and understood. 

To the best of our knowledge, only the recent study by Ma et al. [48] evaluated the oxidative damage in rapeseed (*Brassica napus* L.) plants when grown hydroponically in the presence of 70 and 150 mM NaCl, and the effect of WD on the protection of photosystem II in this crop plant. The results obtained by Ma et al. [48] suggest that WD would have the potential to efficiently reduce Na accumulation in the leaves of plants grown in the presence of 150 mM NaCl. Nevertheless, this is the first study investigating the effectiveness of WD in plants cultivated in soils with extreme salt stress concentrations above 150 mM NaCl. It is generally known that, beyond a concentration of 100 mM NaCl, the phytotoxic effects of salt are increasingly detrimental to the functioning of the biochemical and physiological processes of the lettuce plant [56], initially showing stunted growth, and leaf symptoms such as thickening, wilting, yellowing, discoloration, and dropping, ultimately leading, in the most severe cases, to plant death. 

In this context, we aimed to investigate whether WD could be an effective ally in enhancing the resistance of lettuce plants to high salt stress conditions, (i.e., 200 mM and 300 mM NaCl), since up to date, this research topic has not yet been explored. 

Our results showed that as the NaCl concentration increased, the leaf fresh biomass decreased significantly. This is known to be a sign of stress by the plants since the leaf biomass accumulation is crucial for the survival of the plants [57]. In contrast, the +WD plants were stimulated to produce leaf fresh biomass, except at the highest NaCl concentration (i.e., 300 mM). Wood distillate is to be recognized for its biostimulating action on plant growth, thanks to the presence of various compounds (such as acids, alcohols, aldehydes, ketones, phenols, and nutrients, specifically calcium and iron), that help plants to grow healthily and respond to various environmental stresses [58]. Our results are consistent with those of Ma et al. [48], showing a significant increase in the leaf fresh biomass of rapeseed plants when these plants were foliarly sprayed with 0.2% (*v*/*v*) WD and grown with 150 mM NaCl.

As mentioned above, and also in our case, lettuce plants grown at NaCl concentrations above 200 mM exhibited visible symptoms of leaf thickening, yellowing, and finally leaf loss. The fact that chlorophyll content increased upon exceeding certain salt conditions is not evidence of improved photosynthetic performance by the plants, but a result that corroborates the positive correlation between the increase in measured chlorophyll content and the increase in leaf thickness resulting from exposure to salt stress. 

The uncontrolled uptake of Na ion in salt-enriched soils during plant growth has been identified as a primary factor contributing to the occurrence of Na phytotoxicity phenomenon, causing nutrient imbalances as well as disruptions in plant biochemical and physiological processes [59,60]. Consistent with the results obtained by Ma et al. [48], which a decrease in Na in the leaves of rapeseed plants subjected to growth under NaCl of 70 and 150 mM, we observed a significant reduction in Na by WD only in lettuce leaves exposed to 100 mM NaCl. Therefore, these results might indicate that when the external conditions are no longer favorable for plant growth due to high salt levels, the use of WD could be beneficial because it would seem to help salt-stressed plants to reduce Na accumulation, and thus increase plant tolerance to salt by surviving despite such stressful conditions.

Plants are able to increase the level of carbohydrates to balance the salt-induced osmotic disorders [61]. One of the most common adaptive responses involves a reduction in soluble sugars and, simultaneously, an increase in starch synthesis [62,63,64] to modulate the osmotic potential and cellular turgor [65]. Indeed, our results also revealed that all −WD plants exposed to NaCl, except those grown at the highest NaCl concentration, significantly accumulated starch in the leaves. However, the effect of WD on leaf starch accumulation remains relatively unexplored, as only a few studies have reported a substantial (>100%) increase in starch content in lettuce leaves when sprayed with WD [66].

In this study, we assessed the leaf oxidative stress level through the evaluation of: (i) EL, whose value is linked to the estimation of the integrity of cell plasma membrane; (ii) MDA content, which is correlated to the cell plasma membrane lipid peroxidation level; (iii) ARA, which provides an estimation of the total antioxidant power, and thus of the overall plant capability to scavenge ROS; (iv) TPC, a specific group of powerful plant antioxidants. In particular, we have assisted an increase in EL and MDA for -WD plants as the exposure to NaCl increased, and a decrease in both these parameters for +WD plants only at the two highest NaCl concentrations (i.e., 200 and 300 mM). When these parameters increase, it means that the cell plasma membrane is damaged due to osmotic and ionic stresses (high EL content) or oxidative stress (high MDA content) [67]. For MDA content, our findings are in line with those published by Ma et al. [48], who found a substantial drop in the MDA content of rapeseed plants treated with 0.2% (*v*/*v*) WD at 70 and 150 mM NaCl compared to untreated plants. On the plant antioxidant response capacity, it can be emphasized that salt, in general, induced the activation of the plant response by increasing ARA. However, salt had a great impact in lowering the production and the accumulation of phenolic compounds in lettuce leaves, already noticeable at very low salt concentrations. The actions of WD can be useful in enhancing the non-enzymatic antioxidant compounds to resist not only high salt levels, but also in increasing the levels of phenols in the edible parts of plants grown under non-stressful conditions [68,69]. The use of natural products that induce stress in plants, known as eustress, by activating defense mechanisms and stimulating the increased production of antioxidants [70] is a commonly adopted practice in horticulture to improve the quality of horticultural plants [71]. Wood distillate would also appear to act as an eustressor, strengthening plant defenses and increasing their antioxidant power and the content of antioxidant compounds.

## 4. Materials and Methods

### 4.1. Plant Growth and Treatments

Lettuce (*Lactuca sativa* L., cv. Salanova) seedlings were purchased from a local nursery (Cerretani, Siena, Italy). In our laboratory, seedlings were transplanted into plastic pots (10 × 10 × 10 cm), containing 120 g of a commercial growing medium (VigorPlant Italia srl; the characteristics are as follows: moisture content = 43%; porosity = 92%; pH = 5.30 ± 0.03; EC= 1.12 ± 0.01 mS cm^−1^; and cation exchange capacity = 56.9 ± 2.7 meq100 g DW^−1^). Seedlings were grown for 5 weeks in a climate chamber with a day/night cycle of 16/8 h and 23/20 °C, 60% relative humidity, and 220 μmol s^−1^ m^−2^ PAR (photosynthetically active radiation). Immediately after transplantation, plants were irrigated using different sodium chloride (NaCl) solutions (JT Baker—Fisher Scientific, Milan, Italy) dissolved in water [0 mM (pH: 7.19; EC: 0.61 mS cm^−1^), 50 mM (pH: 7.25; EC: 5.78 mS cm^−1^), 100 mM (pH: 7.34; EC: 11.16 mS cm^−1^), 200 mM (pH: 7.38; EC: 20.60 mS cm^−1^), and 300 mM (pH: 7.42; EC: 29.61 mS cm^−1^)]. These salt concentrations were chosen based on our previous published work [72]. For each of the five treatments, five replicates were prepared. During the experimental growth period, the plants were irrigated with these solutions, when necessary, by maintaining the soil at 70% water holding capacity. Half of the plants from each condition were foliarly sprayed with 0.2% (*v*/*v*) wood distillate (WD), while the remaining half was foliarly sprayed with water once a week for 5 weeks. The WD used (BioDea^®,^ BioEsperia, Arezzo, Italy) was obtained from the pyrolysis process using sweet chestnut (*Castanea sativa* Mill.) pruning as the feedstock. The wood distillate had the physics-chemical characteristics reported in Table 1, already showed in Celletti et al. [38].

At the end of the growth period (corresponding to 5 weeks of growth under the 5 different conditions), the above-ground part of lettuce plants was harvested, weighed for leaf fresh biomass, and stored at −20 °C for subsequent physiological and biochemical analyses.

### 4.2. Leaf Analysis

#### 4.2.1. Chlorophyll

The chlorophyll content was measured using the portable non-destructive device (CCM-300, Opti-Science Inc., Hudson, NH, USA). The measurements were taken, avoiding the nerves of the leaves, on the three youngest and fully expanded leaves [66]. The results were reported as the quantity of chlorophyll on a surface basis (mg m^−2^) [73].

#### 4.2.2. Electrolyte Leakage

Uniform circular sections (Ø 5 mm) of the youngest and fully expanded leaf were obtained by means of a circular stainless-steel tip, and were carefully rinsed three times with deionized water (dH_2_O). Subsequently, the leaf samples were transferred to 50-mL plastic tubes containing 20 mL of dH_2_O and, after 2 h at room temperature, the EC1 of the solutions was measured using a conductivity meter (Crison Basic 30, Crison Instruments, S.A., Barcelona, Spain). Then, the sample tubes were placed in an oven at 90 °C for 25 min. Next, in the cooled solutions, the EC2 was measured for the second time. The following formula was used to calculate the electrolyte leakage (EL) [72]:

EL (%)=(EC1⁄EC2)×100

#### 4.2.3. Sodium

Lettuce leaves were oven-dried at 60 °C and pulverized. Approximately 150 mg DW of this material were microwave-digested (Milestone Ethos 900, Gladeville, Metrohm, Australia) with 3 mL of 70% (*v*/*v*) HNO_3_, 0.2 mL of 50% (*v*/*v*) HF, and 0.5 mL of 30% (*v*/*v*) H_2_O_2_. Inductively coupled plasma-mass spectrometry (ICP-MS, Perkin Elmer NexION 350, Hopkiton, MA, USA) was used to determine the leaf concentration of Na. The analytical quality was assessed using the NCS DC 73350 certified standard reference material, “Poplar leaves”, with recoveries ranging from 96 to 111%. The precision of the analysis was calculated using the coefficient of variation of 5 repetitions and was always >97%. The results are presented on a dry weight basis (g kg _DW_^−1^).

#### 4.2.4. Malondialdehyde

The method proposed by Celletti et al. [74] was used to assess the level of lipid peroxidation, expressed as malondialdehyde (MDA) content, as MDA is considered to be a biomarker of oxidative stress. An amount of 0.5 g FW of lettuce leaves were homogenized in 5 mL of a pre-chilled reagent prepared by dissolving 0.25 g of 2-thiobarbituric acid (TBA) (Merck KGaA, Darmstadt, Germany) in 100 mL of 10% (*w*/*v*) trichloroacetic acid (Panreac, Castellar del Vallès, Barcelona, Spain). The samples were first incubated at 95 °C for 30 min and then ice-cooled to stop the reaction. After centrifugation at 5000 rpm for 20 min, the absorbance of the supernatants was measured at 532 nm and 600 nm using a UV-Vis spectrophotometer (8453, Agilent, Santa Clara, CA, USA). The non-specific turbidity adjustment was derived by subtracting the absorbance value observed at 600 nm. The lipid peroxidation level was estimated using the molar extinction coefficient (155 mM^−1^ cm^−1^) of the MDA–TBA complex.

#### 4.2.5. Carbohydrates

The content of three soluble sugars (fructose, glucose, and sucrose) was determined according to Fedeli et al. [66]. In total, 1 g of the leaf sample was homogenized in 2 mL dH_2_O before a centrifugation step at 15000 rpm for 5 min. The supernatant was filtered at 0.45 µm (LLG-Syringe filters SPHEROS, Petaluma, CA, USA) with a syringe before being analyzed with HPLC (Waters 600 system, Milford, MA, USA), equipped with a Waters 2410 refractive index detector. Sugars were partitioned with dH_2_O as the mobile phase, eluted at 0.5 mL min^−1^, with a Waters Sugar-Pak I ion-exchange column (6.5 × 300 mm) kept at 90 °C using an external temperature controller (Waters Column Heater Module, Milford, MA, USA). Sugar contents were determined using calibration curves prepared by dissolving analytical sugars (Merck KGaA, Darmstadt, Germany) in dH_2_O at a concentration ranging from 0.1 to 20 mg mL^−1^. The results were expressed on a dry weight basis (mg g_DW_^−1^).

The content of the complex carbohydrate, i.e., starch, in the leaf samples was determined using the method described by Loppi et al. [75]. About 50 mg of dried sample was homogenized in 2 mL of dimethyl sulfoxide (DMSO) (≥99.9%, Carlo Erba, Cornaredo, MI, Italy), and then 0.5 mL of 8 M HCl was added. The sample tubes were placed at 60 °C for 30 min and then on ice to stop the reaction. In total, 0.5 mL of 8 M NaOH and 7 mL of dH_2_O was added to the samples. Subsequently, they were centrifuged at 4000 rpm for 5 min. A total of 0.5 mL of supernatant was added to 2.5 mL of Lugol’s solution (previously prepared by mixing 0.05 M HCl, 0.03% (*w*/*v*) I_2_, and 0.06% (*w*/*v*) KI). After 15 min, samples were read at 605 nm using a UV-Vis spectrophotometer (8453, Agilent, Santa Clara, CA, USA). A calibration curve of pure starch (Merck KGaA, Darmstadt, Germany) (in the range of 10–400 g mL^−1^) was used for the quantification of starch in the samples. The results are expressed on a dry weight basis (mg g_DW_^−1^).

#### 4.2.6. Antioxidants

The total antioxidant power was assessed using the method described by Fedeli et al. [76]. After homogenizing 0.5 g FW of lettuce leaves in 2 mL of 80% (*v*/*v*) ethanol, the mixture was centrifuged at 15,000 rpm for 5 min. An aliquot of 200 µL of supernatant was taken and added to 1 mL of 2,2-diphenyl-1-picrylhydrazyl (DPPH) solution (Merck KGaA, Darmstadt, Germany), which was prepared by dissolving 3.9 mg of DPPH in 100 mL of 80% (*v*/*v*) methanol. To quantify the antioxidant capacity of the samples, a blank and a control were prepared. After an incubation of 1 h in the dark, the absorbance of the samples was measured at 517 nm using a UV-Vis spectrophotometer (Agilent 8453, Santa Clara, CA, USA). The total antioxidant power was expressed as the antiradical activity (ARA, %), using the following formula:

ARA (%)=100×[1−(sample absorbance)/(control absorbance)]

The total phenol content (TPC) was determined using the method described by Lamaro et al. [77]. Briefly, 0.5 mg FW of lettuce leaves were homogenized in 4 mL of 70% (*v*/*v*) acetone (Merck KGaA, Darmstadt, Germany). The homogenate was centrifuged at 4000 rpm for 5 min. Afterwards, 0.5 mL of the collected supernatant was added to 3.95 mL dH_2_O, 0.125 mL Folin–Ciocalteu reagent (Merck KGaA, Darmstadt, Germany), 0.75 mL saturated Na_2_CO_3_. After incubation at 37 °C for 30 min, the samples were again centrifuged at 4000 rpm for 5 min and the absorbance of the supernatant was measured at 765 nm using a UV-Vis spectrophotometer (Agilent 8453; Santa Clara, CA, USA). A calibration curve of gallic acid (GA, >98%, Merck KGaA, Darmstadt, Germany) (in the range of 5–20 g mL^−1^) was used for the quantification of TPC in the samples. The results are expressed as mg of the GA equivalent on a dry weight basis (mg GAE g_DW_^−1^).

### 4.3. Statistical Analysis

Due to the non-normality of the data (Shapiro–Wilk test, *p* < 0.05), a non-parametric approach was followed [78]. The parameters analyzed were expressed as their median value and the associated error was expressed as the interquartile range divided by the square root of the number of observations from five biological replicates (n = 5). The significance of differences (*p* < 0.05) between treatments was assessed using the Kruskal–Wallis test and pairwise differences within the same salt treatments were assessed using the Kolmogorov–Smirnov test. All calculations were performed with the R software (v 4.4.0) [79].

## 5. Conclusions

This study revealed the varying thresholds of salt tolerance in lettuce plants, delineating their resilience up to 100 mM NaCl and their subsequent decline in defense mechanisms beyond this threshold. The observed reductions in leaf fresh weight and antioxidant responses, coupled with increased oxidative stress, underscore the critical importance of understanding plant responses to environmental stressors.

In addition, the application of 0.2% (*w*/*w*) WD via foliar spraying emerges as a promising agronomic practice, effectively supporting plant growth and enabling tolerance to extreme NaCl conditions, even from high NaCl concentrations such as 200 mM.

Thus, in the face of worsening environmental challenges, the use of WD is a novel approach that offers a feasible solution for cultivating plants in soils contaminated by high salt levels, traditionally deemed unsuitable, and contributes to sustainable agricultural practices.

## Figures and Tables

**Table 1:** Main physico-chemical characteristics of the wood distillate (Celletti et al. [38]).

Parameter	Value	Method
TOC (% DW)	58.03	CHNS Elemental Analysis
TN (% DW)	1.06	CHNS Elemental Analysis
H (% DW)	7.27	CHNS Elemental Analysis
S (% DW)	0.07	CHNS Elemental Analysis
pH	4	UNI EN ISO 10523:2012
Density (g mL^−1^)	1.05	
Flash point (°C)	>60	ASTM D6450-16a
Total organic compounds (g L^−1^)	33.8	
Acidity (mg L^−1^)	1289	APAT CNR IRSA 2010 B Man 29 2003
Organic acids (mg L^−1^)	32.3	
Acetic acid (mg L^−1^)	21.5	
Polyphenols (g L^−1^)	24.5	
Phenols (g L^−1^)	3	
PCBs (mg L^−1^)	<0.2	CNR IRSA 24b Q 64 Vol 3 1988
Hydrocarbons C < 12 (mg L^−1^)	<0.1	EPA 5021A 2014 + EPA 8015D 2003
Hydrocarbons C10–C40 (mg L^−1^)	<0.1	UNI EN ISO 9377-2:2002
**16 US-EPA PAHs (mg L^−1^)**		EPA 3550C 2007 + EPA 8310 1986
Acenaphthene	<0.05	
Acenaphthylene	<0.05	
Anthracene	<0.05	
Benzo[a]anthracene	<0.05	
Benzo[a]pyrene	<0.05	
Benzo[b]fluoranthene	<0.05	
Benzo[g,h,i]perylene	<0.05	
Benzo[k]fluoranthene	<0.05	
Chrysene	<0.05	
Dibenz[a,h]anthracene	<0.05	
Fluoranthene	<0.05	
Fluorene	<0.05	
Indeno[1,2,3-cd]pyrene	<0.05	
Naphthalene	<0.05	
Phenanthrene	<0.05	
Pyrene	<0.05	
**Macronutrients (mg L^−1^)**		Alkaline melting + ICP-MS analysis
Ca	325.50	
K	23.49	
Mg	6.79	
P	7.28	
**Micronutrients (mg L^−1^)**		Alkaline melting + ICP-MS analysis
Cu	0.18	
Fe	21.16	
Mn	0.58	
Mo	0.0007	
Zn	3.22	
**Other nutrients**		Alkaline melting + ICP-MS analysis
Al	1.96	
Ba	0.06	
Cr	0.03	
Na	103.59	

**TOC**: total organic carbon. **TN**: total nitrogen. **PCBs**: polychlorinated biphenyls. **16 US-EPA PAHs**: list of 16 priority polycyclic aromatic hydrocarbons as classified by the United State Environmental Protection Agency. **Al**: aluminum; **Ba**: barium; **C**: carbon; **Ca**: calcium; **Cr**: chromium; **Cu**: copper; **Fe**: iron; **K**: potassium; **Mg**: magnesium; **Mn**: manganese; **Mo**: molybdenum; **N**: nitrogen; **Na**: sodium; **Zn**: zinc.

## Data Availability

Data will be made available on reasonable request by the corresponding author.
